# The eIF4E2-Directed Hypoxic Cap-Dependent Translation Machinery Reveals Novel Therapeutic Potential for Cancer Treatment

**DOI:** 10.1155/2017/6098107

**Published:** 2017-11-26

**Authors:** Gaelan Melanson, Sara Timpano, James Uniacke

**Affiliations:** Department of Molecular and Cellular Biology, University of Guelph, Guelph, ON, Canada

## Abstract

Hypoxia is an aspect of the tumor microenvironment that is linked to radiation and chemotherapy resistance, metastasis, and poor prognosis. The ability of hypoxic tumor cells to achieve these cancer hallmarks is, in part, due to changes in their gene expression profiles. Cancer cells have a high demand for protein synthesis, and translational control is subsequently deregulated. Various mechanisms of translation initiation are active to improve the translation efficiency of select transcripts to drive cancer progression. This review will focus on a noncanonical cap-dependent translation initiation mechanism that utilizes the eIF4E homolog eIF4E2, a hypoxia-activated cap-binding protein that is implicated in hypoxic cancer cell migration, invasion, and tumor growth in mouse xenografts. A historical perspective about eIF4E2 and its various aliases will be provided followed by an evaluation of potential therapeutic strategies. The recent successes of disabling canonical translation and eIF4E with drugs should highlight the novel therapeutic potential of targeting the homologous eIF4E2 in the treatment of hypoxic solid tumors.

## 1. Introduction

The initiation step of protein synthesis is a focal point of translational control (reviewed in [1]). The mammalian target of rapamycin complex 1 (mTORC1) is a master regulator of this process and senses several external stimuli such as nutrients and oxygen to control cell proliferation [2, 3]. The first step of translation involves the binding of the heterotrimeric eukaryotic initiation factor 4F (eIF4F) complex to the 5′ cap of mRNA. Specifically, it is eIF4E that is the cap-binding component of eIF4F (reviewed in [4]). The mTORC1 regulates the first step of translation by phosphorylating and inactivating the inhibitor of eIF4E, 4E-binding protein (4EBP), under normal conditions [5–7]. When oxygen is low, for example, the kinase activity of mTORC1 is repressed and 4EBP binds to and sequesters eIF4E [8–10]. Several cap-independent mechanisms exist to translate key mRNAs required to overcome a specific stress such as internal ribosomal entry sites (IRES) [11, 12] and upstream open reading frames (uORFs) [13, 14]. It is important to note that the “who's who” of cancer-driving mutations occurs in upstream regulators of mTORC1 (e.g., Akt [15], PTEN [16], PI3K [17], and Ras [18]), which uncouple this master regulator from sensing nutrient and oxygen deprivation. This constitutively active mTORC1 causes eIF4E-driven translation to be hyperactive in most cancers and is currently a major target of cancer therapeutics (reviewed in [19]). This literature review will focus on an alternative cap-dependent translation mechanism that utilizes the eIF4E homolog eIF4E2 [20], a cap-binding protein that is part of a metastatic gene signature [21] and required for tumor growth in mouse xenografts [22]. This pathway is activated by hypoxia [23], a characteristic of the microenvironment common to many solid tumors. A historical perspective about eIF4E2 will be provided followed by an evaluation of potential therapeutic strategies.

## 2. Translation Initiation

### 2.1. Canonical Translation Initiation

**Figure 1:**
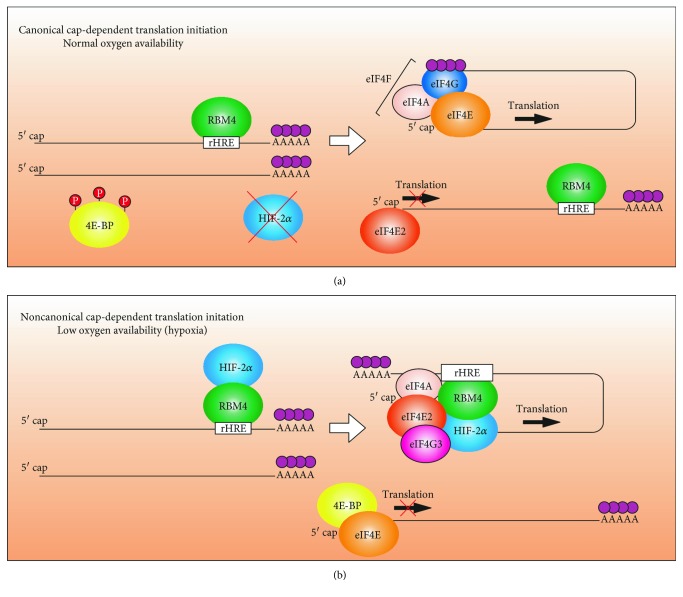
Model of canonical and noncanonical cap-dependent translation initiation. (a) Canonical cap-dependent translation mediated by eIF4E. Under normoxic conditions, the 4E-binding protein (4EBP) is phosphorylated by mTORC1 and repressed, allowing eIF4E to bind the 5′ cap of mRNA, eIF4G, and eIF4A forming the eIF4F complex to initiate translation. HIF-2*α* is degraded in the presence of oxygen and is unavailable to recruit eIF4E2 to the 5′ cap of transcripts containing RNA hypoxia response elements (rHREs) in their 3′ UTR. RBM4 is an RNA-binding protein that recognizes a motif in the rHRE and is essential for the translation of these transcripts in the presence of HIF-2*α*. (b) Under hypoxic conditions, HIF-2*α* is stabilized and interacts with RBM4 to recruit eIF4E2 to the 5′ cap of rHRE-containing transcripts independent of the poly(A)-tail. The eIF4E2 interacts with eIF4G3 and eIF4A to form a hypoxic eIF4F complex (eIF4F^H^) to initiate the translation of rHRE-containing transcripts. The 4EBP is hypophosphorylated, binds to eIF4E, and blocks the eIF4G binding site to repress canonical cap-dependent translation.

Eukaryotic translation efficiency is heavily reliant on posttranscriptional modifications to the mRNA at the 3′ end (poly(A)-tail) and at the 5′ end (7-methyl-guanosine triphosphate cap; m^7^GTP) [24, 25]. Canonical cap-dependent translation initiation begins with and can be regulated at two separate events. First, GTP-bound eIF2 binds to the initiator methionyl-tRNA (Met-tRNAi) and then to the 40S ribosomal subunit (reviewed in [4]). The eIF1, eIF3, eIF5, and eIF5B associate followed by the formation of the 43S preinitiation complex (PIC). The other event is the binding of eIF4F onto the 5′ m^7^GTP cap of mRNA (Figure 1(a)). eIF4F is a heterotrimeric complex composed of the cap-binding protein eIF4E, the scaffold protein eIF4G, and the RNA helicase eIF4A. The assembly of this complex is the primary target of regulatory proteins such as the 4EBPs, as the cellular availability of eIF4E controls the switch between canonical cap-dependent translation and noncanonical cap-dependent translation or cap-independent mechanisms [26] (Figure 1).

### 2.2. Cap-Independent Translation Initiation

Hypoxia results in a decreased rate of protein synthesis; yet, de novo proteins are still expressed despite the repression of eIF4F-mediated translation [23]. IRES sequences were first discovered in the poliovirus and encephalomyocarditis virus RNA genomes [11, 27], but a growing number of cellular IRES-containing transcripts are being identified with the potential to be translated via cap-independent mechanisms [12]. It is important to note, however, that the existence of cellular IRES-containing transcripts remains controversial due to the technical challenges associated with their detection (reviewed in [28, 29]). Some classic examples of cellular IRES-containing transcripts are those encoding the mouse hypoxia-inducible factor- (HIF-) 1*α* [30] and vascular endothelial growth factor (VEGF) [31]. Both of these proteins are central to the cellular response to hypoxia: HIF-1*α* is the oxygen-regulated subunit of the HIF-1 heterodimer that mediates the transcriptional response to hypoxia, and VEGF plays a key role in blood vessel formation (angiogenesis). While it is possible for the IRES to independently interact with the 40S and 60S ribosomal subunits, canonical translation factors (e.g., eIF4A and eIF3) and IRES-transacting factors (ITAFs) are usually involved to enhance translation efficiency. To ensure selective translation of the appropriate transcript, many ITAFs are expressed in a tissue-specific manner (reviewed in [32]). Importantly, IRES-mediated translation accounts for less than 1% of hypoxic protein synthesis [33]. This observation may have been, in part, explained through the identification of an alternative noncanonical cap-dependent translation machinery that is activated in hypoxic cells and mediated by the eIF4E homolog, eIF4E2 [23].

## 3. eIF4E2

### 3.1. Discovery, Function, and Regulation

eIF4E2 (also known as 4E homologous protein (4EHP)) was first identified in a human fetal brain cDNA library as 30% identical and 60% similar to eIF4E [20]. In humans, the *EIF4E2* gene is located on the long arm of chromosome 2 and its tissue distribution is ubiquitous albeit at 10-fold lower levels than eIF4E [34]. The peptide sequence of eIF4E2 is highly conserved with eIF4E in a core region, possessing two Trp ➔ Tyr (Trp43 and Trp56) substitutions within the eight evolutionarily conserved tryptophan residues of eIF4E. eIF4E2 facilitates 5′ cap binding via *π*–*π* stacking between the aromatic rings of Tyr78 and Trp124 at 100-fold lower affinity than eIF4E [35]. Structural analysis identified the reduced affinity of eIF4E2 for the 5′ cap resulted from mutation of Arg162 that forms a stabilizing hydrogen bond with the *β*-phosphate of the mRNA cap in eIF4E [20]. Additionally, eIF4E2 interacts weakly or not at all with 4EBP and eIF4G, respectively [20, 36]. Structural variation around the eIF4G/4E-BP docking sequence, (S/T)VXXFW, impairs the ability of these proteins to associate with eIF4E2. Therefore, due to its inability to bind eIF4G, eIF4E2 inhibits translation when bound to the 5′ cap. Some of the first studies describing the cellular function of eIF4E2 were performed in the developing *Drosophila* embryo. The *Drosophila* homolog of eIF4E2 (d4EHP) inhibits the anterior translation of maternal *caudal* mRNA [37] and represses *belle* mRNA translation in the ovary [38]. Translation repression of *hunchback* mRNA in the posterior of the embryo involves eIF4E2 [39], but it is not obligatory [40]. In mice, eIF4E2 inhibits the translation of *Hoxb4* mRNA in female germ cells [41] and acts as a translation repressor in a complex with GIGYF2 that is essential for normal development in mice [42, 43]. In humans, eIF4E2 also forms a complex with GIGYF2 and/or 4E-T to repress translation initiation [42, 44, 45]. Interestingly, while eIF4E2 binds to the transporter 4E-T, it does not require it to shuttle to the nucleus as does eIF4E suggesting a different role for eIF4E2 in the nucleus [46]. The E3 ubiquitin ligase HHARI interacts to polyubiquitylate eIF4E2, and the authors of this study speculate that this interaction may alter its cap-binding affinity [47].

Several lines of evidence suggest that human eIF4E2 has a distinct cytoplasmic role in the stress response. During periods of arsenite or actinomycin D treatment, eIF4E2 is not sequestered to stress granules or P-bodies, respectively, like its homolog eIF4E [46]. During interferon, genotoxic stress, and pathogen infection, the ubiquitin-like molecule ISG15 is covalently added to eIF4E2 to increase its cap-binding affinity [48]. In mice with defects in glycogen storage, *EIF4E2* (named eIF4EL3 in this study) is one of 44 genes to significantly vary by more than 1.5-fold by increasing 1.57-fold. Conversely, in mice that hyperaccumulate glycogen, *EIF4E2* levels decrease by 2.08-fold [49]. This suggests that eIF4E2 could have an inverse relationship with energy availability, although the biological significance has not been explored. When normal human fibroblasts experience microgravity stress during space flight in cell culture, *EIF4E2* is one of 50 genes that are upregulated [50]. In mice given a 20 min treatment of forebrain ischemia, *EIF4E2* is one of 25 genes in the hippocampus that display a greater than 3-fold transcriptional increase (6-fold) [51]. Finally, eIF4E2 is required for development, a process driven by hypoxia, as knockouts in both *Drosophila* [52] and mouse [42] are embryonic lethal. These data present a theme where eIF4E2 becomes available in the cytoplasm and increases in mRNA and protein abundance in response to various forms of stress.

In 2012, eIF4E2 was identified as an activator of translation initiation during periods of hypoxia [23]. This finding came from the observation of HIF-2*α*-dependent, but transcription-independent, hypoxic accumulation of the epidermal growth factor receptor (EGFR) [23]. HIF-2*α* was found to interact with RNA-binding motif 4 (RBM4) in the 3′ UTR of hundreds of transcripts containing RNA hypoxia response elements (rHREs) (Figure 1(b)). The HIF-2*α*/RBM4 complex joins the 3′ UTR to the 5′ cap via eIF4E2, but not eIF4E, independent of the poly(A)-binding proteins [23]. The eIF4E2 interacts with eIF4A and eIF4G3 to form a hypoxic eIF4F (eIF4F^H^) complex that increases translation efficiency independent of mRNA abundance [53] (Figure 1(b)). Interestingly, the protein levels of eIF4E2, eIF4G3, and eIF4A do not change in hypoxia relative to normoxia [23, 53] suggesting that posttranslational modifications or compartmentalization may play a role in modifying their activities. On the other hand, HIF-2*α* is essential for eIF4E2 activity [23]. Therefore, HIF-2*α* could be the sole activator of eIF4E2 in hypoxia. Since eIF4E2 appears to play a role in the response to other stresses in several eukaryotes, as mentioned above, it is tempting to speculate that each stress induces unique activators of eIF4E2. However, the role of eIF4E2 in other stresses besides hypoxia has yet to be explored in humans. The eIF4E2 is a strong candidate for a general stress response translation factor that is independent of mTORC1 regulation due to the inability to bind, or weak binding, to 4EBP [20, 36]. Further investigation will be required to elucidate the mechanisms of translation initiation and regulation of eIF4F^H^ activity.

### 3.2. Nomenclature and Conservation across Eukaryotes

The protein encoded by the human *EIF4E2* gene can be found in the literature under several aliases including 4E homologous protein (4EHP), 4E-like protein (4E-LP), eIF4EL3, and eIF4E2. This inconsistency in nomenclature for human eIF4E2 may have caused some delays or barriers in data dissemination because of the following: (1) One must know to search through the literature using all of the eIF4E2 aliases to gain access to all the available information. (2) Several studies misleadingly state or imply that eIF4E2 is part of the canonical translation initiation apparatus, especially those where the *EIF4E2* gene that appears in a big data set under a different alias such as microarray or high-throughput sequencing [21, 49–51, 54].

The *EIF4E2* gene is expressed as a divergent homolog of eIF4E in most eukaryotes. This review has already discussed reports of eIF4E2 function in mammals (humans and mice). In addition, several studies have reported functions for eIF4E2 in model organisms such as *Drosophila melanogaster* [37–42, 52], *Caenorhabditis elegans* [55], *Schizosaccharomyces pombe* [56, 57], and *Arabidopsis thaliana* [58]. In *Drosophila*, eIF4E2 is most commonly referred to as 4EHP and is involved in translation repression during the development of the embryo [37–42, 52]. In *C. elegans*, the eIF4E2 homolog is named IFE-4 and is involved in the translation initiation of a subset of mRNAs mostly required for egg laying [55]. In *S. pombe*, the eIF4E2 protein is part of the translation initiation machinery and is required to resist nutrient, salt, and temperature stress, and the eIF4E:eIF4E2 ratio shifts from 2 : 1 at low temperature (15°C) to 1 : 5 at high temperature (42°C) [56, 57]. Similar to human eIF4E2, *S. pombe* eIF4E2 binds very poorly to eIF4G (>100-fold less than eIF4E) [56]. In *A. thaliana*, the eIF4E2 homolog is named novel cap-binding protein (nCBP) and it can initiate the translation of a subset of mRNAs [58]. Therefore, eIF4E2 appears to be conserved across eukaryotes and is often found to be involved in selective translation repression or activation during stress or development. Humans express a third member of the eIF4E family, eIF4E3, albeit with a much more limited tissue distribution [34]. The eIF4E3 binds m^7^GTP in an atypical manner [59], marginally suppresses eIF4E-dependent translation in diffuse large B-cell lymphoma [60], and is a tumor suppressor [59]. This review will not discuss eIF4E3 any further as there are much fewer studies relative to eIF4E and eIF4E2.

### 3.3. eIF4E2 in Cancer

The inconsistency in human eIF4E2 nomenclature has likely played a role in delaying the dissemination of its connection to cancer. In 2003, the metastatic potential of multiple tumor types could be predicted by a six-gene signature that contained *EIF4E2* (named eIF4EL3 in this study) [21]. In 2011, an examination was performed to 105 patients with metastatic non-small-cell lung carcinoma (NSCLC) for single-nucleotide polymorphisms (SNPs) that change the rate of overall survival during treatment with paclitaxel and carboplatin chemotherapeutics [54]. The SNP that produced the biggest effect was in individuals homozygous for an A ➔ G mutation in the third exon of *EIF4E2*. These individuals had a significantly lower rate of overall survival (*p* = 8.4 × 10^−8^, hazard ratio = 4.22 (confidence interval: 2.32–7.66)) of 7.7 months compared to 18 months for individuals who were heterozygous or homozygous for the A allele. Additionally, expression of *EIF4E2* was found to be significantly increased in metastatic NSCLC tumors. However, the authors incorrectly stated in this study that the *EIF4E2* gene encodes eIF4E. Finally, a detection method for disorders of the lung involving transcriptomic profiling was patented in 2005 that describes changes in *EIF4E2* transcript levels (named eIF4EL3 in the patent) as a marker [61].

It was not until the response of the eIF4E2 protein to hypoxia was described at the molecular level in 2012 [23] that studies began emerging examining its role in tumor growth. The connection to cancer progression was not surprising considering the identity of the eIF4E2 mRNA targets identified through PAR-CLIP [23]. Dozens of eIF4E2 mRNA targets have strong ties to cancer such as a group of receptor tyrosine kinases including EGFR, platelet-derived growth factor receptor-alpha, insulin-like growth factor 1 receptor, and HER-2, which most cancers overexpress at least one. In 2014, several cancer cell lines stably depleted for eIF4E2 (U87MG glioblastoma, 786-O renal cell carcinoma, and HCT116 colorectal carcinoma) displayed impaired proliferation and increased apoptosis only in hypoxia [22]. Moreover, eIF4E2-depleted xenografts in mice displayed significantly less growth than controls. The eIF4E2 was shown to participate in active translation in hypoxic mouse xenografts, and the growth of established tumors in mice could be halted or reversed by treatment with shRNA targeting *EIF4E2* [22]. In 2017, *CDH22* mRNA was identified as a hypoxic eIF4E2 target that encodes cadherin-22, a cell-cell adhesion molecule providing cancer cells with collective migratory and invasive properties specifically in hypoxia [62]. Furthermore, CDH22 expression colocalized with hypoxic regions in human glioma and breast cancer patient tumor specimens and high protein levels significantly correlated with tumor size, cancer stage, and progression-free survival [62]. Thus, proteins synthesized via eIF4E2 offer the possibility of being prognostic markers of hypoxia in cancer patients and eIF4E2, an attractive therapeutic target to disable the adaptation of cells to the hypoxic tumor microenvironment.

## 4. Hypoxia

### 4.1. Hypoxia Inducible Factors

HIFs are the master regulators of the transcriptional response to hypoxia in the cell. These heterodimeric transcription factors are strongly tied to cancer progression and consist of an oxygen-regulated *α*-subunit (HIF-1*α* or HIF-2*α*) and a constitutively expressed *β*-subunit (HIF-1*β*) (reviewed in [63, 64]). The stabilization of both HIFs leads to angiogenesis, metabolic reprogramming, immortalization, evasion of apoptosis, migration and invasion, generation of cancer stem cells, and chemo- and radiotherapy resistance. While there is overlap in HIF-1*α* and HIF-2*α* structure and function [65, 66], these two *α*-subunits can have distinct roles in the cell. HIF-1*α* and HIF-2*α* share most of their transcription targets but have been shown to bind to distinct targets as well [67]. Interestingly, HIF-*α* homologs display unexpected suppressive interactions, with enhanced expression of HIF-2*α* suppressing HIF-1*α* and vice versa [67]. The *α*-subunits also display temporal differences in their expression. Analysis of neuroblastoma cell lines showed that HIF-1*α* protein levels peak within 2 h of hypoxic exposure and then steadily decreased [68]. In contrast, HIF-2*α* protein expression peaks and remains constant at ≥24 h of hypoxia and is more abundant than HIF-1*α* under physiological oxygen conditions (37 mmHg or 5% O_2_) [68]. *In vivo*, HIF-1*α* is expressed in all mammalian tissues and cell types [69], and HIF-2*α* expression was initially characterized as restricted to specific cell types, including developing blood vessels and the lung [70]. However, exposure of rats to hypoxia causes HIF-2*α* to accumulate in all organs investigated, including the brain, heart, lung, kidney, liver, pancreas, and intestine [71]. Therefore, HIF-1*α* is likely involved in acute responses to *in vitro* hypoxia and HIF-2*α* with chronic hypoxia. It is important to note that *in vivo*, HIF-1*α* is expressed in chronically hypoxic tumor regions, which could be partly due to genetic events or oxygen-independent stabilization by several stimuli present in the tumor microenvironment (reviewed in [72]).

The HIF-*α*-subunits each have nontranscriptional roles in the cell independent of interactions with HIF-1*β*. HIF-1*α* inhibits firing of replication origins, decreases DNA replication, and induces cell cycle arrest in various cell types through binding to Cdc6 [73]. HIF-2*α* binds to RBM4 in the 3′ UTR rHRE of select transcripts as part of the eIF4E2-directed hypoxic translation machinery (Figure 1(b)), while HIF-1*α* does not play a role in this process [23]. There had been previous reports of HIF-2*α*, not HIF-1*α*, becoming trapped in the cytoplasm upon chronic hypoxic exposure [74, 75], which is consistent with a role in translation. It is important to note that HIF-2*α* does not preferentially bind to mRNAs that are induced by HIF-dependent transcription [23]. This suggests that transcription and translation are distinct layers of regulation for the hypoxic gene expression response.

### 4.2. Hypoxic Regulation of Translation Initiation

Regulatory proteins are in place to regulate translation initiation by disrupting either the assembly/loading of the PIC complex or the assembly/cap-binding activity of eIF4F (introduced in section 2.1). These two modes of regulation occur as a biphasic response to hypoxia (acute and chronic) mediated through two distinct pathways. Under endoplasmic reticulum stress induced by acute hypoxic conditions, the kinase PERK phosphorylates eIF2*α* preventing its association with Met-tRNAi and loading of the PIC, thus repressing canonical cap-dependent translation (reviewed in [76]). However, under chronic hypoxia, eIF2*α* begins to dephosphorylate and a second pathway emerges to maintain translation repression through disruption of eIF4F and sequestration of eIF4E in the cytoplasm by the 4EBPs and in the nucleus by the transporter 4E-T [77]. During chronic hypoxia, mTORC1 is impaired in its ability to phosphorylate the 4EBPs, which allows them to bind to eIF4E using a similar binding motif as eIF4G (YxxxxL*ϕ*) (reviewed in [4, 78]). The binding of the 4E-BPs will not interfere with eIF4E cap-binding ability but will inhibit eIF4G association and canonical cap-dependent mRNA translation (Figure 1). Under chronic hypoxic exposure, cells also induce the expression of REDD1, which decreases the phosphorylation of ribosomal S6 kinase, another mTORC1 phosphotarget, indicating that REDD1 is involved in regulating mTORC1 during hypoxia. Likewise, the presence of the tuberous sclerosis complex heterodimer, which is necessary for downregulating mTORC1 activity, is induced by REDD1 activity in response to hypoxia [9]. In normal cells under chronic hypoxia, the ability to load the PIC is regained through dephosphorylation of eIF2*α* (reviewed in [76]). Repression of eIF4E allows for mTORC1-independent mechanisms to take over, such as noncanonical cap-dependent translation (eIF4E2 driven) or cap-independent processes. It is important to note that hypoxic regulation of translation is uncoupled in cancer [79] due to the frequently mutated upstream regulators of mTORC1 (e.g., Akt [15], PTEN [16], PI3K [17], and Ras [18]). Constitutively active mTORC1 causes eIF4E-driven translation to be hyperactive in most cancers and is currently a major target of cancer therapeutics (reviewed in [19]). However, evidence suggests eIF4E2 as a significant contributor for cancer cells to display various cancer hallmarks, for tumor growth, and as a possible predictor of metastasis and poor outcome [21, 22, 54, 62]. Targeting eIF4E2 relative to eIF4E has the potential to be more selective for malignant cells (hypoxic tumor cells) subsequently leading to lower toxicity.

## 5. Targeting eIF4F and eIF4F^H^ in Cancer

**Figure 2:**
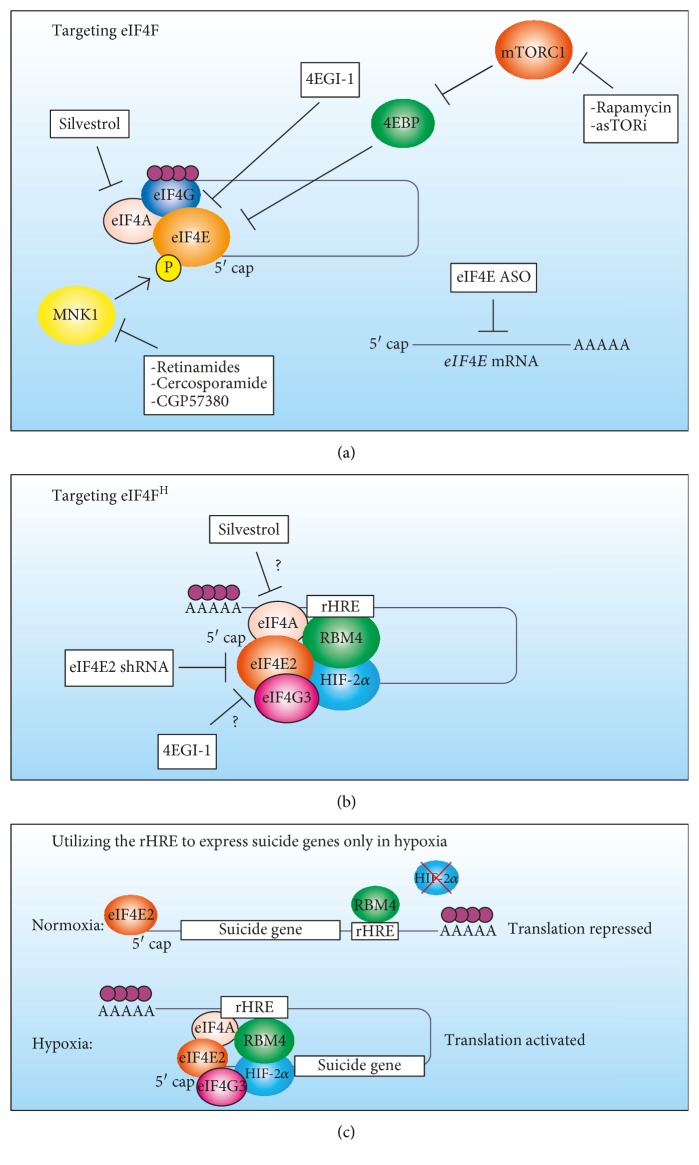
Summary of current therapeutic strategies targeting eIF4F and possible therapeutic interventions for eIF4F^H^. (a) Rapamycin and asTORi inhibit mTORC1, allowing hypophosphorylated 4E-binding protein (4EBP) to block the eukaryotic initiation factor 4E (eIF4E) binding site from eIF4G. Inhibitors that degrade Mnk1 kinase prevent eIF4E phosphorylation, which reduces tumor growth. The most promising therapeutics includes eIF4E suppression via antisense oligonucleotides (ASOs) and disrupting the eIF4E-eIF4G interaction with drugs such as 4EGI-1. Inhibiting the eIF4A RNA helicase has displayed high preclinical potency, especially silvestrol, in mouse models of tumor progression. (b) Current evidence demonstrates that suppression of eIF4E2 via lentiviral-delivered shRNAs is effective at stalling or reversing tumor growth in mouse xenografts of several different cancer cell lines. Drugs used to target eIF4F such as 4EGI-1 and silvestrol could potentially also inhibit eIF4F^H^ through blocking the eIF4E2-eIF4G3 interaction or inhibiting eIF4A, respectively. (c) The 3′ UTR RNA hypoxia response element (rHRE) that is found in eIF4E2-dependent transcripts could be exploited as a hypoxia-inducible RNA sequence. The rHRE would repress synthesis until the therapeutic RNA reaches the hypoxic tumor cells. This would be especially useful when paired with a suicide gene, for example.

It is evident that an increase in eIF4E activity is oncogenic due to the many ways this is achieved in cancer cells such as gene duplication, increased *EIF4E* transcription, and increased eIF4E availability due to constitutive mTORC1 activation (reviewed in [80]). Phosphorylation of eIF4E via Mnk1 increases its activity [81], and this event is common in various cancers to drive their progression [82–84]. Several therapeutic strategies have been developed that either interfere with mTORC1 or eIF4E (reviewed in [19]). Rapamycin and several analogs are specific inhibitors of mTORC1 that have been extensively used in the clinic or in clinical trials but have shown a lower than expected efficacy [85]. More potent inhibitors of mTORC1 have been developed, such as asTORi [85], but many tumors have displayed resistance through a high eIF4E/4EBP ratio [86] or a switch to mTORC1-independent translation [87] (Figure 2(a)). Inhibitors that degrade Mnk1 kinase and prevent eIF4E phosphorylation have shown promise in breast cancer cell lines [88] (Figure 2(a)). Many efforts are ongoing to directly target eIF4E or the assembly of the eIF4F complex in various preclinical and clinical trials (reviewed in [19]). The most promising therapeutics includes eIF4E suppression via antisense oligonucleotides [89, 90] and disrupting the eIF4E-eIF4G interaction with drugs such as 4EGI-1 [91, 92] (Figure 2(a)). Targeting translation has the same rationale as the classic target of cancer therapeutics and cell proliferation: cancer cells proliferate more, therefore requiring more mRNA translation. However, proliferation and translation are fundamental processes that normal healthy cells utilize. The dosage for therapeutics targeting these pathways must be carefully considered as there could be a fine line between killing a cancer cell and a healthy cell. Targeting eIF4E2-driven translation could be more selective to cancer cells, or at least hypoxic tumor cells, rather than healthy cells because chronic hypoxia is associated with disease.

Development of therapeutics targeting eIF4E2 is in their infancy as this cap-binding protein has only recently been linked to tumor growth and its mechanisms of initiation and regulation are only beginning to be elucidated. Current evidence demonstrates that suppression of eIF4E2 via lentiviral-delivered shRNAs is effective at stalling or reversing tumor growth in mouse xenografts of several different cancer cell lines [22] (Figure 2(b)). Importantly, oxygenated cells are unaffected while hypoxic cells display widespread cell death. It will be a priority that interventions targeting eIF4E2 hit the metastatic and/or progressive phenotype, and not just cancer cells. Therefore, while eIF4E2 is involved in tumor growth [22] and several cancer cell hallmarks *in vitro* [22, 62], future studies should aim to more tightly link eIF4E2 to high-risk, metastatic cancer disease.

The eIF4E2 is part of the eIF4F^H^ complex that includes an eIF4G homolog, eIF4G3 [53]. Therefore, effective strategies that disrupt eIF4E-eIF4G interactions could be employed in a similar fashion with eIF4E2-eIF4G3 (Figure 2(b)). Interestingly, eIF4A is also part of eIF4F^H^, and drugs inhibiting this RNA helicase have displayed high preclinical potency, especially silvestrol, in mouse models of tumor progression [93]. Part of the reason that this drug is so effective could be that it disrupts both eIF4E- and eIF4E2-dependent translations (Figure 2(b)). Because of the high sequence homology between *EIF4E* and *EIF4E2*, some posttranslational regulatory pathways could be shared and therapeutically exploited such as phosphorylation.

Besides disrupting eIF4E2 activity or complex formation, the 3′ UTR rHRE that is found in eIF4E2-dependent transcripts could be exploited as a hypoxia-inducible RNA sequence. The hypoxia response elements found within the promoters of HIF target genes have been used in gene therapy for cancer treatment [94, 95], but RNA has emerged as an attractive source of gene products in place of DNA [96]. mRNA has several advantages including a lack of requirement for nuclear entry, which poses a barrier to plasmid DNA delivery, especially in nondividing or slowly dividing hypoxic cells. mRNA also has a negligible chance of integrating into the host genome avoiding aberrant transcription of oncogenes. A major limitation of gene therapy is selective expression, and an rHRE fusion could achieve this. There is also evidence that the rHRE is not only strictly an activator of hypoxic translation but also a repressor of translation in oxygenated conditions [23]. Therefore, the rHRE could repress synthesis until the therapeutic RNA reaches the hypoxic tumor region. This would be especially useful when paired with a suicide gene, for example (Figure 2(c)). Whether developing a small molecule inhibitor of eIF4E2 or an rHRE-RNA fusion, targeting hypoxia has its challenges such as accessing hypoxic areas that are remote from blood vessels and impaired uptake in hypoxic cells (reviewed in [97]). Constant improvements are being made in nanomedicine and drug design to generate tumor-reaching vehicles [98] and hypoxia-activated bioreductive prodrugs (reviewed in [97]), respectively.

## 6. Physioxia

Another important factor to consider when investigating cellular responses to hypoxia and targeting them with drugs is physiological oxygen levels (physioxia). The partial oxygen pressure within various human organs *in vivo* is much lower than it is in the atmosphere [99, 100]. By the time oxygen enters the lungs and is distributed throughout the various tissues, its availability is well below 21% (160 mmHg). Each tissue has its own “normoxia,” and for this reason, the term “physioxia” is used to more effectively describe the *in vivo* partial oxygen pressure. The mean partial pressure of oxygen range reported by Carreau et al. is 29.2 ± 1.8 mmHg in the muscle to 72 ± 20 mmHg in the kidney (or the equivalent of 3.8 ± 0.2% O_2_ to 9.5 ± 2.6% O_2_) [99]. The oxygen within cells and organelles could be even lower due to consumption rates. In human cell culture, measures are taken to control the cellular environment to better reflect physiological conditions such as temperature and pH. Oxygen is a surprisingly neglected variable as cells are routinely cultured in ambient air (21% O_2_). Furthermore, it is important to consider that 24 h is required for the dissolved oxygen in culture media to equilibrate with the ambient air [101]. Therefore, cell culture studies in low oxygen incubators should be performed after a 24 h exposure of either the cells or the media alone (before adding it to the cells) to the new oxygen environment.

**Figure 3:**
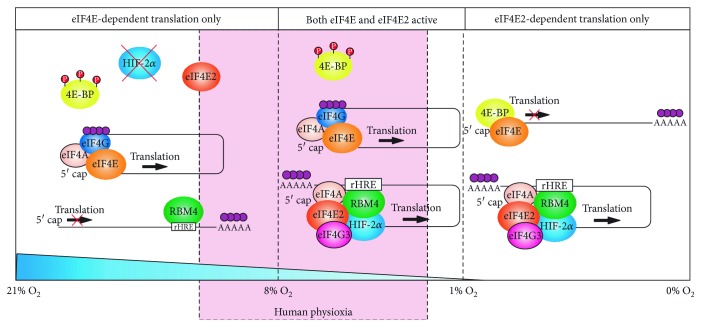
Both eIF4E- and eIF4E2-dependent translation initiations are active in the range of human physiological tissue oxygenation. The mean partial pressure of oxygen in human tissues (physioxia) ranges from 29.2 ± 1.8 mmHg in the muscle to 72 ± 20 mmHg in the kidney (or the equivalent of 3.8 ± 0.2% O_2_ to 9.5 ± 2.6% O_2_). The ability of mTORC1 to phosphorylate and inactivate the eIF4E repressor 4EBP decreases significantly between 1 and 3% O_2_. This allows eIF4E-dependent translation to remain active between 1 and 21% O_2_. Furthermore, the eIF4E2 hypoxic activator HIF-2*α* is stabilized below 8% O_2_, allowing eIF4E2-dependent translation to be active in the low to midrange of human physioxia. Therefore, there is a window during physiological tissue oxygenation where eIF4E and eIF4E2 are cooperating to produce the cellular proteome. It is important to note that to achieve the indicated oxygen concentrations in cell culture media, 24 h is required for the dissolved oxygen to equilibrate with the ambient air.

HIF-2*α*, but not HIF-1*α*, is stabilized under chronic 5% O_2_ [68], which overlaps with the mean tissue oxygenation of several organs. Indeed, HIF-2*α* activates eIF4E2-directed translation in several primary cell lines at oxygen levels as high as 5–8% O_2_ evidenced by eIF4E2 and rHRE-containing transcripts associated with polysomes [102] (Figure 3). The eIF4E and some of its most dependent transcripts (e.g., TOP-containing mRNAs [103]) are associated with polysomes at oxygen levels as low as 1–3% O_2_ in primary cells [102]_._ This suggests that eIF4E2 is actively participating in translation in the low range of physioxia, while eIF4E is active throughout the entire range. Moreover, this provides the intriguing possibility that there is a window within the physiological range of oxygen availability where both eIF4E and eIF4E2 are contributing to the cellular proteome through interactions with distinct mRNAs. Cancer cells displayed a shifted window of dual eIF4E and eIF4E2 usage (3–12% O_2_) suggesting that eIF4E2 is activated and eIF4E is sequestered at higher oxygen levels relative to primary cells [102]. Perhaps during gradual tumor hypoxification, there is selective pressure on hypoxic cells to repress eIF4E-dependent translation early and activate eIF4E2 early. Indeed, studies have shown that overexpressing eIF4E selectively disables hypoxic tumor cells [104] and eIF4E2 mRNA targets are enriched in cancer-driving genes [22, 23, 53, 62, 102].

The implications of the above studies are twofold. First, eIF4E2-dependent translation may be important in the normal physiology of human tissues with mean partial oxygen pressures in the low range of physioxia such as the brain and muscle. Several classic and modern cancer therapeutics target essential processes such as cell proliferation and protein synthesis, but eIF4E2 initially emerged as a potential drug target that could be more selective to cancer cells. Therefore, the possibility that small molecule targeting of eIF4E2 and fusing rHRE sequences to suicide genes are toxic to at least some tissues must be noted. Second, if a fundamental process like protein synthesis is differentially regulated in physioxia relative to the common cell culture condition of normoxia (21% O_2_), then perhaps, cancer therapeutics in general should be tested in conditions of lower oxygen.

## 7. Conclusions

We present a summary of evidence leading to the discovery and ongoing characterization of noncanonical cap-dependent hypoxic translation and its involvement in tumor growth. This review consolidates studies using several different eIF4E2 aliases to highlight that it is conserved across eukaryotes and could have a role in advanced cancer stage in humans. The role of eIF4E2 in various model organisms suggests that it participates in the general stress response with perhaps stress-specific activators. In human cells, eIF4E2 is part of a hypoxic eIF4F complex (eIF4F^H^) with eIF4G3 and eIF4A that increases the translation efficiency of mRNAs irrespective of their abundance. However, further efforts are needed to fully elucidate the mechanism of initiation through interactions with either canonical initiation factors or their homologs and to more tightly link eIF4E2 to high-risk, metastatic cancer disease. The recent successes of disabling canonical translation and eIF4E with drugs should highlight the novel therapeutic potential of targeting the homologous eIF4E2 in the treatment of hypoxic solid tumors.

## References

[1] Gebauer F., Hentze M. W. (2004). Molecular mechanisms of translational control. *Nature Reviews Molecular Cell Biology*.

[2] Dowling R. J., Topisirovic I., Alain T. (2010). mTORC1-mediated cell proliferation, but not cell growth, controlled by the 4E-BPs. *Science*.

[3] Wullschleger S., Loewith R., Hall M. N. (2006). TOR signaling in growth and metabolism. *Cell*.

[4] Sonenberg N., Hinnebusch A. G. (2009). Regulation of translation initiation in eukaryotes: mechanisms and biological targets. *Cell*.

[5] Pause A., Belsham G. J., Gingras A. C. (1994). Insulin-dependent stimulation of protein synthesis by phosphorylation of a regulator of 5′-cap function. *Nature*.

[6] Brunn G. J., Hudson C. C., Sekulić A. (1997). Phosphorylation of the translational repressor PHAS-I by the mammalian target of rapamycin. *Science*.

[7] Lin T. A., Kong X., Haystead T. (1994). PHAS-I as a link between mitogen-activated protein kinase and translation initiation. *Science*.

[8] Braunstein S., Karpisheva K., Pola C. (2007). A hypoxia-controlled cap-dependent to cap-independent translation switch in breast cancer. *Molecular Cell*.

[9] Brugarolas J., Lei K., Hurley R. L. (2004). Regulation of mTOR function in response to hypoxia by REDD1 and the TSC1/TSC2 tumor suppressor complex. *Genes & Development*.

[10] Liu L., Cash T. P., Jones R. G., Keith B., Thompson C. B., Simon M. C. (2006). Hypoxia-induced energy stress regulates mRNA translation and cell growth. *Molecular Cell*.

[11] Pelletier J., Sonenberg N. (1988). Internal initiation of translation of eukaryotic mRNA directed by a sequence derived from poliovirus RNA. *Nature*.

[12] Weingarten-Gabbay S., Elias-Kirma S., Nir R. (2016). Systematic discovery of cap-independent translation sequences in human and viral genomes. *Science*.

[13] PD L., Harding H. P., Ron D. (2004). Translation reinitiation at alternative open reading frames regulates gene expression in an integrated stress response. *The Journal of Cell Biology*.

[14] Vattem K. M., Wek R. C. (2004). Reinitiation involving upstream ORFs regulates *ATF4* mRNA translation in mammalian cells. *Proceedings of the National Academy of Sciences of the United States of America*.

[15] Carpten J. D., Faber A. L., Horn C. (2007). A transforming mutation in the pleckstrin homology domain of AKT1 in cancer. *Nature*.

[16] Li J., Yen C., Liaw D. (1997). *PTEN*, a putative protein tyrosine phosphatase gene mutated in human brain, breast, and prostate cancer. *Science*.

[17] Samuels Y., Wang Z., Bardelli A. (2004). High frequency of mutations of the *PIK3CA* gene in human cancers. *Science*.

[18] Forbes S. A., Bindal N., Bamford S. (2011). COSMIC: mining complete cancer genomes in the catalogue of somatic mutations in cancer. *Nucleic Acids Research*.

[19] Bhat M., Robichaud N., Hulea L., Sonenberg N., Pelletier J., Topisirovic I. (2015). Targeting the translation machinery in cancer. *Nature Reviews. Drug Discovery*.

[20] Rom E., Kim H. C., Gingras A. C. (1998). Cloning and characterization of 4EHP, a novel mammalian eIF4E-related cap-binding protein. *The Journal of Biological Chemistry*.

[21] Ramaswamy S., Ross K. N., Lander E. S., Golub T. R. (2003). A molecular signature of metastasis in primary solid tumors. *Nature Genetics*.

[22] Uniacke J., Perera J. K., Lachance G., Francisco C. B., Lee S. (2014). Cancer cells exploit eIF4E2-directed synthesis of hypoxia response proteins to drive tumor progression. *Cancer Research*.

[23] Uniacke J., Holterman C. E., Lachance G. (2012). An oxygen-regulated switch in the protein synthesis machinery. *Nature*.

[24] Gallie D. R. (1991). The cap and poly(A) tail function synergistically to regulate mRNA translational efficiency. *Genes & Development*.

[25] Shatkin A. J. (1976). Capping of eucaryotic mRNAs. *Cell*.

[26] Svitkin Y. V., Herdy B., Costa-Mattioli M., Gingras A. C., Raught B., Sonenberg N. (2005). Eukaryotic translation initiation factor 4E availability controls the switch between cap-dependent and internal ribosomal entry site-mediated translation. *Molecular and Cellular Biology*.

[27] Jang S. K., Kräusslich H. G., Nicklin M. J., Duke G. M., Palmenberg A. C., Wimmer E. (1988). A segment of the 5′ nontranslated region of encephalomyocarditis virus RNA directs internal entry of ribosomes during in vitro translation. *Journal of Virology*.

[28] Kozak M. (2001). New ways of initiating translation in eukaryotes?. *Molecular and Cellular Biology*.

[29] Kozak M. (2003). Alternative ways to think about mRNA sequences and proteins that appear to promote internal initiation of translation. *Gene*.

[30] Lang K. J., Kappel A., Goodall G. J. (2002). Hypoxia-inducible factor-1α mRNA contains an internal ribosome entry site that allows efficient translation during normoxia and hypoxia. *Molecular Biology of the Cell*.

[31] Stein I., Itin A., Einat P., Skaliter R., Grossman Z., Keshet E. (1998). Translation of vascular endothelial growth factor mRNA by internal ribosome entry: implications for translation under hypoxia. *Molecular and Cellular Biology*.

[32] Holcik M., Sonenberg N. (2005). Translational control in stress and apoptosis. *Nature Reviews Molecular Cell Biology*.

[33] Young R. M., Wang S. J., Gordan J. D., Ji X., Liebhaber S. A., Simon M. C. (2008). Hypoxia-mediated selective mRNA translation by an internal ribosome entry site-independent mechanism. *The Journal of Biological Chemistry*.

[34] Joshi B., Cameron A., Jagus R. (2004). Characterization of mammalian eIF4E-family members. *European Journal of Biochemistry*.

[35] Zuberek J., Kubacka D., Jablonowska A. (2007). Weak binding affinity of human 4EHP for mRNA cap analogs. *RNA*.

[36] Tee A. R., Tee J. A., Blenis J. (2004). Characterizing the interaction of the mammalian eIF4E-related protein 4EHP with 4E-BP1. *FEBS Letters*.

[37] Cho P. F., Poulin F., Cho-Park Y. A. (2005). A new paradigm for translational control: inhibition via 5′-3′ mRNA tethering by Bicoid and the eIF4E cognate 4EHP. *Cell*.

[38] Yarunin A., Harris R. E., Ashe M. P., Ashe H. L. (2011). Patterning of the Drosophila oocyte by a sequential translation repression program involving the d4EHP and Belle translational repressors. *RNA Biology*.

[39] Cho P. F., Gamberi C., Cho-Park Y. A., Cho-Park I. B., Lasko P., Sonenberg N. (2006). Cap-dependent translational inhibition establishes two opposing morphogen gradients in *Drosophila* embryos. *Current Biology*.

[40] Weidmann C. A., Goldstrohm A. C. (2012). *Drosophila* Pumilio protein contains multiple autonomous repression domains that regulate mRNAs independently of Nanos and brain tumor. *Molecular and Cellular Biology*.

[41] Villaescusa J. C., Buratti C., Penkov D. (2009). Cytoplasmic Prep1 interacts with 4EHP inhibiting *Hoxb4* translation. *PLoS One*.

[42] Morita M., Ler L. W., Fabian M. R. (2012). A novel 4EHP-GIGYF2 translational repressor complex is essential for mammalian development. *Molecular and Cellular Biology*.

[43] Fu R., Olsen M. T., Webb K., Bennett E. J., Lykke-Andersen J. (2016). Recruitment of the 4EHP-GYF2 cap-binding complex to tetraproline motifs of tristetraprolin promotes repression and degradation of mRNAs with AU-rich elements. *RNA*.

[44] von Stechow L., Typas D., Carreras Puigvert J. (2015). The E3 ubiquitin ligase ARIH1 protects against genotoxic stress by initiating a 4EHP-mediated mRNA translation arrest. *Molecular and Cellular Biology*.

[45] Chapat C., Jafarnejad S. M., Matta-Camacho E. (2017). Cap-binding protein 4EHP effects translation silencing by microRNAs. *Proceedings of the National Academy of Sciences of the United States of America*.

[46] Kubacka D., Kamenska A., Broomhead H., Minshall N., Darzynkiewicz E., Standart N. (2013). Investigating the consequences of eIF4E2 (4EHP) interaction with 4E-transporter on its cellular distribution in HeLa cells. *PLoS One*.

[47] Tan N. G., Ardley H. C., Scott G. B., Rose S. A., Markham A. F., Robinson P. A. (2003). Human homologue of ariadne promotes the ubiquitylation of translation initiation factor 4E homologous protein, 4EHP. *FEBS Letters*.

[48] Okumura F., Zou W., Zhang D. E. (2007). ISG15 modification of the eIF4E cognate 4EHP enhances cap structure-binding activity of 4EHP. *Genes & Development*.

[49] Parker G. E., Pederson B. A., Obayashi M., Schroeder J. M., Harris R. A., Roach P. J. (2006). Gene expression profiling of mice with genetically modified muscle glycogen content. *Biochemical Journal*.

[50] Liu Y., Wang E. (2008). Transcriptional analysis of normal human fibroblast responses to microgravity stress. *Genomics, Proteomics & Bioinformatics*.

[51] Nagata T., Takahashi Y., Sugahara M. (2004). Profiling of genes associated with transcriptional responses in mouse hippocampus after transient forebrain ischemia using high-density oligonucleotide DNA array. *Brain Research. Molecular Brain Research*.

[52] Valzania L., Ono H., Ignesti M. (2016). *Drosophila* 4EHP is essential for the larval-pupal transition and required in the prothoracic gland for ecdysone biosynthesis. *Developmental Biology*.

[53] Ho J. J., Wang M., Audas T. E. (2016). Systemic reprogramming of translation efficiencies on oxygen stimulus. *Cell Reports*.

[54] Sato Y., Yamamoto N., Kunitoh H. (2011). Genome-wide association study on overall survival of advanced non-small cell lung cancer patients treated with carboplatin and paclitaxel. *Journal of Thoracic Oncology*.

[55] Dinkova T. D., Keiper B. D., Korneeva N. L., Aamodt E. J., Rhoads R. E. (2005). Translation of a small subset of *Caenorhabditis elegans* mRNAs is dependent on a specific eukaryotic translation initiation factor 4E isoform. *Molecular and Cellular Biology*.

[56] Ptushkina M., Berthelot K., von der Haar T., Geffers L., Warwicker J., McCarthy J. E. (2001). A second eIF4E protein in *Schizosaccharomyces pombe* has distinct eIF4G-binding properties. *Nucleic Acids Research*.

[57] Ptushkina M., Malys N., McCarthy J. E. (2004). eIF4E isoform 2 in *Schizosaccharomyces pombe* is a novel stress-response factor. *EMBO Reports*.

[58] Ruud K. A., Kuhlow C., Goss D. J., Browning K. S. (1998). Identification and characterization of a novel cap-binding protein from *Arabidopsis thaliana*. *The Journal of Biological Chemistry*.

[59] Osborne M. J., Volpon L., Kornblatt J. A., Culjkovic-Kraljacic B., Baguet A., Borden K. L. B. (2013). eIF4E3 acts as a tumor suppressor by utilizing an atypical mode of methyl-7-guanosine cap recognition. *Proceedings of the National Academy of Sciences of the United States of America*.

[60] Landon A. L., Muniandy P. A., Shetty A. C. (2014). MNKs act as a regulatory switch for eIF4E1 and eIF4E3 driven mRNA translation in DLBCL. *Nature Communications*.

[61] Brody J. S., Spira A. (2005). *Detection Methods for Disorders of the Lung*.

[62] Kelly N. J., Varga J. F. A., Specker E. J., Romeo C. M., Coomber B. L., Uniacke J. (2017). Hypoxia activates cadherin-22 synthesis via eIF4E2 to drive cancer cell migration, invasion and adhesion. *Oncogene*.

[63] Majmundar A. J., Wong W. J., Simon M. C. (2010). Hypoxia-inducible factors and the response to hypoxic stress. *Molecular Cell*.

[64] Schito L., Semenza G. L. (2016). Hypoxia-inducible factors: master regulators of cancer progression. *Trends Cancer*.

[65] Loboda A., Jozkowicz A., Dulak J. (2010). HIF-1 and HIF-2 transcription factors—similar but not identical. *Molecules and Cells*.

[66] Ratcliffe P. J. (2007). HIF-1 and HIF-2: working alone or together in hypoxia?. *The Journal of Clinical Investigation*.

[67] Raval R. R., Lau K. W., Tran M. G. B. (2005). Contrasting properties of hypoxia-inducible factor 1 (HIF-1) and HIF-2 in von Hippel-Lindau-associated renal cell carcinoma. *Molecular and Cellular Biology*.

[68] Holmquist-Mengelbier L., Fredlund E., Löfstedt T. (2006). Recruitment of HIF-1α and HIF-2α to common target genes is differentially regulated in neuroblastoma: HIF-2α promotes an aggressive phenotype. *Cancer Cell*.

[69] Wiener C. M., Booth G., Semenza G. L. (1996). *In vivo*expression of mRNAs encoding hypoxia-inducible factor 1. *Biochemical and Biophysical Research Communications*.

[70] Tian H., McKnight S. L., Russell D. W. (1997). Endothelial PAS domain protein 1 (EPAS1), a transcription factor selectively expressed in endothelial cells. *Genes & Development*.

[71] Wiesener M. S., Jürgensen J. S., Rosenberger C. (2003). Widespread hypoxia-inducible expression of HIF-2α in distinct cell populations of different organs. *The FASEB Journal*.

[72] Semenza G. L. (2003). Targeting HIF-1 for cancer therapy. *Nature Reviews. Cancer*.

[73] Hubbi M. E., Kshitiz D. M. G., Rey S. (2013). A nontranscriptional role for HIF-1α as a direct inhibitor of DNA replication. *Science Signaling*.

[74] Park S. K., Dadak A. M., Haase V. H., Fontana L., Giaccia A. J., Johnson R. S. (2003). Hypoxia-induced gene expression occurs solely through the action of hypoxia-inducible factor 1α (HIF-1α): role of cytoplasmic trapping of HIF-2α. *Molecular and Cellular Biology*.

[75] Talks K. L., Turley H., Gatter K. C. (2000). The expression and distribution of the hypoxia-inducible factors HIF-1α and HIF-2α in normal human tissues, cancers, and tumor-associated macrophages. *The American Journal of Pathology*.

[76] Baird T. D., Wek R. C. (2012). Eukaryotic initiation factor 2 phosphorylation and translational control in metabolism. *Advances in Nutrition*.

[77] Koritzinsky M., Magagnin M. G., van den Beucken T. (2006). Gene expression during acute and prolonged hypoxia is regulated by distinct mechanisms of translational control. *The EMBO Journal*.

[78] Topisirovic I., Svitkin Y. V., Sonenberg N., Shatkin A. J. (2011). Cap and cap-binding proteins in the control of gene expression. *Wiley Interdiscip Rev RNA*.

[79] Connolly E., Braunstein S., Formenti S., Schneider R. J. (2006). Hypoxia inhibits protein synthesis through a 4E-BP1 and elongation factor 2 kinase pathway controlled by mTOR and uncoupled in breast cancer cells. *Molecular and Cellular Biology*.

[80] Silvera D., Formenti S. C., Schneider R. J. (2010). Translational control in cancer. *Nature Reviews. Cancer*.

[81] Pyronnet S., Imataka H., Gingras A. C., Fukunaga R., Hunter T., Sonenberg N. (1999). Human eukaryotic translation initiation factor 4G (eIF4G) recruits mnk1 to phosphorylate eIF4E. *The EMBO Journal*.

[82] Furic L., Rong L., Larsson O. (2010). eIF4E phosphorylation promotes tumorigenesis and is associated with prostate cancer progression. *Proceedings of the National Academy of Sciences of the United States of America*.

[83] Robichaud N., del Rincon S. V., Huor B. (2015). Phosphorylation of eIF4E promotes EMT and metastasis via translational control of SNAIL and MMP-3. *Oncogene*.

[84] Topisirovic I., Ruiz-Gutierrez M., Borden K. L. (2004). Phosphorylation of the eukaryotic translation initiation factor eIF4E contributes to its transformation and mRNA transport activities. *Cancer Research*.

[85] Benjamin D., Colombi M., Moroni C., Hall M. N. (2011). Rapamycin passes the torch: a new generation of mTOR inhibitors. *Nature Reviews. Drug Discovery*.

[86] Alain T., Sonenberg N., Topisirovic I. (2012). mTOR inhibitor efficacy is determined by the eIF4E/4E-BP ratio. *Oncotarget*.

[87] Muranen T., Selfors L. M., Worster D. T. (2012). Inhibition of PI3K/mTOR leads to adaptive resistance in matrix-attached cancer cells. *Cancer Cell*.

[88] Ramalingam S., Gediya L., Kwegyir-Afful A. K. (2014). First MNKs degrading agents block phosphorylation of eIF4E, induce apoptosis, inhibit cell growth, migration and invasion in triple negative and Her2-overexpressing breast cancer cell lines. *Oncotarget*.

[89] DeFatta R. J., Nathan C. O., De Benedetti A. (2000). Antisense RNA to eIF4E suppresses oncogenic properties of a head and neck squamous cell carcinoma cell line. *Laryngoscope*.

[90] Graff J. R., Konicek B. W., Vincent T. M. (2007). Therapeutic suppression of translation initiation factor eIF4E expression reduces tumor growth without toxicity. *The Journal of Clinical Investigation*.

[91] Chen L., Aktas B. H., Wang Y. (2012). Tumor suppression by small molecule inhibitors of translation initiation. *Oncotarget*.

[92] Moerke N. J., Aktas H., Chen H. (2007). Small-molecule inhibition of the interaction between the translation initiation factors eIF4E and eIF4G. *Cell*.

[93] Cencic R., Carrier M., Galicia-Vázquez G. (2009). Antitumor activity and mechanism of action of the cyclopenta[*b*]benzofuran, silvestrol. *PLoS One*.

[94] Binley K., Iqball S., Kingsman A., Kingsman S., Naylor S. (1999). An adenoviral vector regulated by hypoxia for the treatment of ischaemic disease and cancer. *Gene Therapy*.

[95] Ruan H., Wang J., Hu L., Lin C. S., Lamborn K. R., Deen D. F. (1999). Killing of brain tumor cells by hypoxia-responsive element mediated expression of BAX. *Neoplasia*.

[96] Kormann M. S., Hasenpusch G., Aneja M. K. (2011). Expression of therapeutic proteins after delivery of chemically modified mRNA in mice. *Nature Biotechnology*.

[97] Wilson W. R., Hay M. P. (2011). Targeting hypoxia in cancer therapy. *Nature Reviews. Cancer*.

[98] Shi J., Kantoff P. W., Wooster R., Farokhzad O. C. (2017). Cancer nanomedicine: progress, challenges and opportunities. *Nature Reviews. Cancer*.

[99] Carreau A., El Hafny-Rahbi B., Matejuk A., Grillon C., Kieda C. (2011). Why is the partial oxygen pressure of human tissues a crucial parameter? Small molecules and hypoxia. *Journal of Cellular and Molecular Medicine*.

[100] Harrison D. K., Vaupel P. (2014). Heterogeneity in tissue oxygenation: from physiological variability in normal tissues to pathophysiological chaos in malignant tumours. *Advances in Experimental Medicine and Biology*.

[101] Newby D., Marks L., Lyall F. (2005). Dissolved oxygen concentration in culture medium: assumptions and pitfalls. *Placenta*.

[102] Timpano S., Uniacke J. (2016). Human cells cultured under physiological oxygen utilize two cap-binding proteins to recruit distinct mRNAs for translation. *The Journal of Biological Chemistry*.

[103] Thoreen C. C., Chantranupong L., Keys H. R., Wang T., Gray N. S., Sabatini D. M. (2012). A unifying model for mTORC1-mediated regulation of mRNA translation. *Nature*.

[104] Rouschop K. M., Dubois L., Schaaf M. B. E. (2011). Deregulation of cap-dependent mRNA translation increases tumour radiosensitivity through reduction of the hypoxic fraction. *Radiotherapy and Oncology*.

